# Preparation and Properties of Gel Polymer Electrolytes with Li_1.5_Al_0.5_Ge_1.5_(PO_4_)_3_ and Li_6.46_La_3_Zr_1.46_Ta_0.54_O_12_ by UV Curing Process

**DOI:** 10.3390/polym16040464

**Published:** 2024-02-07

**Authors:** Xinghua Liang, Qiankun Hun, Lingxiao Lan, Bing Zhang, Zhikun Chen, Yujiang Wang

**Affiliations:** 1 Guangxi Key Laboratory of Automobile Components and Vehicle Technology, Guangxi University of Science & Technology, Liuzhou 545006, China; lxh304@126.com (X.L.); 13383371921@163.com (Q.H.); 13152528815@163.com (Y.W.); 2 Liuzhou Wuling Automobile Industry Co., Ltd., Liuzhou 545006, China; 3 Foshan Taoyuan Advanced Manufacturing Research Institute, Foshan 528225, China; 13067887972@163.com

**Keywords:** gel polymer electrolytes, interfacial stability, thermal stability, UV curing process, LAGP, LLZTO

## Abstract

Poly (vinylidene fluoride-co-hexafluoropropylene) (PVDF-HFP)-based gel polymer electrolytes (GPEs) are considered a promising electrolyte candidate for polymer lithium-ion batteries (LIBs) because of their free-standing shape, versatility, security, flexibility, lightweight, reliability, and so on. However, due to problems such as low ionic conductivity, PVDF-HFP can only be used on a small scale when used as a substrate alone. To overcome the above shortcomings, GPEs were designed and synthesized by a UV curing process by adding NASICON-type Li_1.5_Al_0.5_Ge_1.5_(PO_4_)_3_ (LAGP) and garnet-type Li_6.46_La_3_Zr_1.46_Ta_0.54_O_12_ (LLZTO) to PVDF-HFP. Experimentally, GPEs with 10% weight LLZTO in a PVDF-HFP matrix had an ionic conductivity of up to 3 × 10^−4^ S cm^−1^ at 25 °C. When assembled into LiFePO_4_/GPEs/Li batteries, a discharge-specific capacity of 81.5 mAh g^−1^ at a current density of 1 C and a capacity retention rate of 98.1% after 100 cycles at a current density of 0.2 C occurred. Therefore, GPEs added to LLZTO have a broad application prospect regarding rechargeable lithium-ion batteries.

## 1. Introduction

In recent years, lithium-ion batteries (LIBs) have become increasingly important in daily life due to their high energy density and safety factors [1]. Almost all commercial LIBs use thermally unstable liquid electrolytes that have reached the energy density limit; thus, it is becoming increasingly essential to develop LIBs with higher energy density and better safety [2]. Solid polymer electrolytes (SPEs) have received much attention due to their high safety and ease of handling [3,4]. However, due to the low ionic conductivity and narrow electrochemical window of SPEs at room temperature, their development in LIBs is limited [5]. Gel polymer electrolytes (GPEs) were created to address these issues. GPEs can reduce the loss of liquid electrolytes while maintaining the ionic conduction properties of the liquid with low internal impedance and high mobility to carry a charge [6,7]; as a result, they have attracted the attention of a wide range of scholars in recent years.

GPEs combine a liquid electrolyte’s diffusion properties with a solid electrolyte’s cohesiveness. Liquid electrolytes are typically composed of lithium salts and organic solvents. At the same time, polymers in solids mainly include polyacrylonitrile (PAN) [8,9], polymethyl methacrylate (PMMA) [10,11], polyethylene oxide (PEO) [12,13], polyvinylidene fluoride (PVDF) [14,15], and polyvinylidene fluoride-hexafluoropropylene (PVDF-HFP) [3,16]. PVDF is considered a reasonable polymer matrix in polymer systems due to its high dielectric constant. However, PVDF has highly structured molecular chains, which give it a glass transition temperature (Tg) and crystallinity that are not conducive to the solid electrolyte, thus reducing ionic conductivity. In contrast, hexafluoropropylene (HFP) has a heterogeneous phase, and PVDF can copolymerize with it to form PVDF-HFP [17]. PVDF-HFP has relatively low Tg and crystallinity and better thermal and electrochemical stability at room temperature, attributed to specific functional groups and strong electron-withdrawing effects in PVDF-HFP [18]. Although the performance of GPEs based on PVDF-HFP is better than that of PVDF, it still needs to be improved. A general method to enhance the performance of GPEs is to add fillers to the polymer matrix, which are divided into two types. The first one is inert fillers, such as SiO_2_ [19,20], TiO_2_ [21,22], etc. Adding inert fillers will inhibit the crystallization of the polymer, reduce the crystallinity, make the polymer segment movement more active, and thus improve the ionic conductivity; The other is reactive fillers, such as Li_0.3_La_0.557_TiO_3_ (LLZO) [2,23], Li_6.75_La_3_Zr_1.75_Ta_0.25_O_12_(LLZTO) [24,25], Li_1+x_Al_x_Ti_2−x_(PO_4_)_3_ (LATP) [26,27], Li_1.5_Al_0.5_Ge_1.5_(PO_4_)_3_ (LAGP) [28,29], etc. Due to the presence of lithium-ion in these materials, ion conduction can be carried out, thereby improving ionic conductivity.

UV curing polymerization systems can be cured under photocatalytic conditions, bearing the advantages of fast curing speeds and mild curing conditions. In recent years, researchers have conducted in-depth analyses on photopolymerization, the curing depth, and the curing process. At the same time, phase change materials of UV curing polymers have also been applied to 3D printing, providing more research ideas for the preparation of complex structures and the diversified applications of shape-stabilized phase change materials.

In this paper, GPEs were designed and synthesized by the UV curing process, and the effects of no filler or filler (LAGP and LLZTO) on GPEs were compared. The total ionic conductivity of LAGP is 3.38 × 10^−4^ S cm^−1^ [30], and the total ionic conductivity of LLZTO is 6.9 × 10^−4^ S cm^−1^ [31]. In cycle tests, it was experimentally proven that adding LLZTO resulted in higher ionic conductivity, higher discharge-specific capacity at high current density, and higher capacity retention. Experiments showed that it has a broad application prospect in the next generation of high-performance LIBs.

## 2. Materials and Methods

### 2.1. Preparation of LAGP and LLZTO

LAGP was obtained by solid-phase synthesis [32]. Lithium carbonate (Li_2_O_3_, AR Merck KGaA, Darmstadt, Germany), aluminum hydroxide (Al (OH)_3_, AR, Merck KGaA, Germany), germanium oxide (GeO_2_, 99.99%, Merck KGaA, Germany), and ammonium dihydrogen phosphate (NH_4_H_2_PO_4_, AR, Merck KGaA, Germany) were mixed well with a planetary mill. The mixture was heated in an alumina crucible at 700 °C for 2 h. The resulting powder was reground and sintered at 1350 °C for another 2 h and annealed at 500 °C for 2 h, followed by natural cooling to obtain LAGP. After synthesis, LAGP had a particle size of 0.5 μm.

LLZTO was obtained by solid-phase synthesis [33]. The appropriate amounts of lanthanum oxide (La_2_O_3_, AR, Merck KGaA, Germany), zirconium dioxide (ZrO_2_, AR, Merck KGaA, Germany), and titanium dioxide (TiO_2_, AR, Merck KGaA, Germany) were weighed. Then, lithium carbonate (Li_2_CO_3_, AR, Merck KGaA, Germany) was weighed with a stoichiometric ratio of more than 10%. These powders were mixed with ethanol and a planetary mill ball mill was used for 24 h. Then, excess Li_2_CO_3_ was added to make up for the loss of lithium in the synthesis process. The resulting precursor was sintered in a muffle furnace at a temperature of 900 °C and kept for 10 h to obtain LLZTO. After synthesis, LLZTO had a particle size of 0.5 μm.

### 2.2. Preparation of Gel Polymer Electrolytes

**Figure 1:**
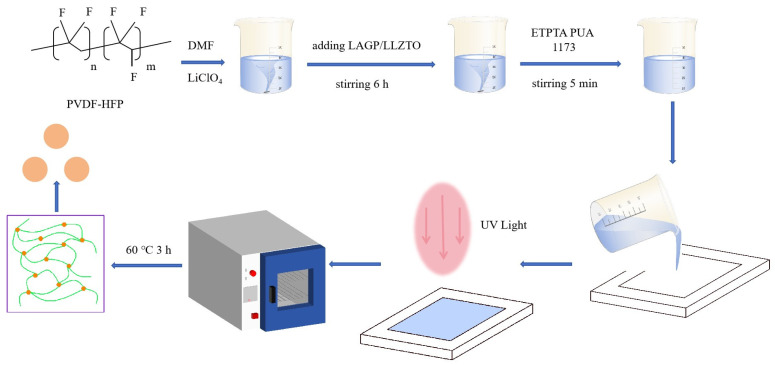
Schematic diagram for the fabrication process of the gel polymer electrolytes (GPEs).

GPEs were prepared for the UV curing process. The preparation flow chart is shown in Figure 1. PVDF-HFP (PVDF-HFP, Mn = 600,000, Arkema, Colombes, France), LAGP, and LLZTO were put into a vacuum drying oven for later use. The magnetic stirrer was turned on and adjusted to 60 °C. Then, the DMF (Macklin Shanghai, China) was weighed in a beaker and put on the magnetic stirrer. PVDF-HFP (25 wt. % relative to DMF) was added to the beaker, and the mouth of the cup was sealed with a sealing film until complete dissolution of the matrix. Then, LiClO_4_ (Macklin, Shanghai, China), LAGP, or LLZTO (weight ratio to PVDF-HFP was 0, 5, 10, 15, 20 wt. %, respectively) were stirred in the beaker for 6 h. Subsequently, plasticizers trimethylolpropane ethoxylate triacrylate (ETPTA, Mn = 428, Macklin, Shanghai, China), polyurethane acrylate (PUA, RYOJI, Frankfurt, Germany), and 2-Hydroxy-2-methylpropiophenone (1173, Chembridge, Beijing, China) were added to it. The magnetic stirrer was turned off after five minutes of stirring, and remained at room temperature for five minutes. Then, the preparations were poured into the mold to make them evenly distributed, and then dried in a drying oven for 3 h to obtain PVDF-HFP/LAGP or PVDF-HFP/LLZTO films, with 0, 5, 10, 15, and 20 wt. %. GPEs prepared by LAGP or LLZTO for PVDF-HFP were recorded as PVDF-HFP-LiClO_4_, PLA 5%, PLA 10%, PLA 15%, PLA 20% or PVDF-HFP-LiClO_4_, PLL 5%, PLL 10%, PLL 15%, PLL 20%, respectively. The dried film reached a circular shape (19 mm diameter) and was immediately transferred to an Ar-filled glove box (both with a concentration of less than 0.1 ppm of water and oxygen). When assembling the battery, a trace amount of liquid electrolyte (1 M LiPF6 in EC: DMC: EMC = 1:1:1 Vol% with 1% VC) was dropped to make the interface wettable.

### 2.3. Characterization and Testing Methods for GPEs

The prepared GPEs were analyzed by X-ray diffractometer (XRD, DX-2700, Dandong, China, Cu-Kα, 40 kV × 30 mA). Surface and cross-section analyses of GPEs were carried out using scanning electron microscopy (SEM, Phenom spectra G2, Shanghai, China). The tensile strength of GPEs was tested by a universal testing machine (WDW-5, Tenson, Jinan, China). Thermogravimetric analysis (TGA) was performed in a nitrogen atmosphere using a TG analysis system (NetzschF3Tarsus, Bayern, Germany) from 30 to 800 °C with a heating rate of 10 °C min^−1^. Fourier transform infrared (FTIR, Spectrum 100, PerkinElmer, Waltham, MA, USA) was used to determine the functional groups on the surface of GPEs prepared at room temperature using spectroscopy in the range of 400~3500 cm^−1^.

### 2.4. Electrochemical Properties

The electrochemical properties of GPEs were evaluated by an electrochemical workstation (DH7000, Donghua, Jingjiang, China) at room temperature. In particular, electrochemical impedance spectroscopy (EIS) was performed on an open-circuit voltage (OCV) of 1 MHz to 10 MHz with an amplitude of 10 mV. At the same time, the ionic conductivity of GPEs was determined with two closed stainless steel (SS) electrodes. The ionic conductivity (σ) was calculated according to Equation (1):

(1) $$\sigma =\frac{L}{RS}$$

where σ is the ionic conductivity of the GPEs (S cm^−1^), *L* is the thickness of the GPEs (cm), *R* is the volume ohmic resistance (Ω), and *S* is the contact area between the GPEs and the blocking SS electrode (cm^2^). The activation energy *E*_*a*_ of the reaction was calculated by the Arrhenius equation (Equation (2)):

(2) $$\sigma =A\exp\left[-\frac{E_{a}}{RT}\right]$$

where *A* is a pre-exponential constant, *E*_*a*_ is the activation energy (eV), *R* is the universal gas constant (J K^−1^ mol^−1^), and *T* is the temperature (K) during the test.

At the same time, symmetrical Li/GPEs/Li batteries were assembled, the lithium-ion mobility number (*t*_*Li*_^+^) of GPEs was determined by EIS and DC polarization methods, and *t*_*Li*_^+^ was calculated according to Equation (3):

(3) $$t_{{Li}^{+}}=\frac{I_{s}\left(∆V-R_{0}I_{0}\right)}{I_{0}\left(∆V-R_{s}I_{s}\right)}$$

where *t*_*Li*_^+^ is the number of lithium-ion mobility; *I*_0_, *I*_s_, *R*_0_, and *R*_s_ are the initial current, steady-state polarization current, initial resistance, and steady-state resistance, respectively; and Δ*V* is the applied polarization potential of 10 mV.

The rate and cycle test of the battery was carried out on the battery test system (Neware, Dongguan, China). Commercial LiFePO_4_ (LFP, Macklin, Shanghai, China) was used as the positive active material, and lithium sheets were used as the negative electrode without further modification. LFP, PVDF, and conductive carbon were weighed with a mass ratio of 8:1:1. Then, they were mixed evenly in the solvent, the obtained slurry was put on aluminum foil, they were dried in a vacuum drying oven for 36 h, and then cut into discs with a diameter of 14 mm for later use. The assembled LFP/GPEs/Li batteries were tested in the voltage range of 2.8 to 4.0 V.

## 3. Results

**Figure 2:**
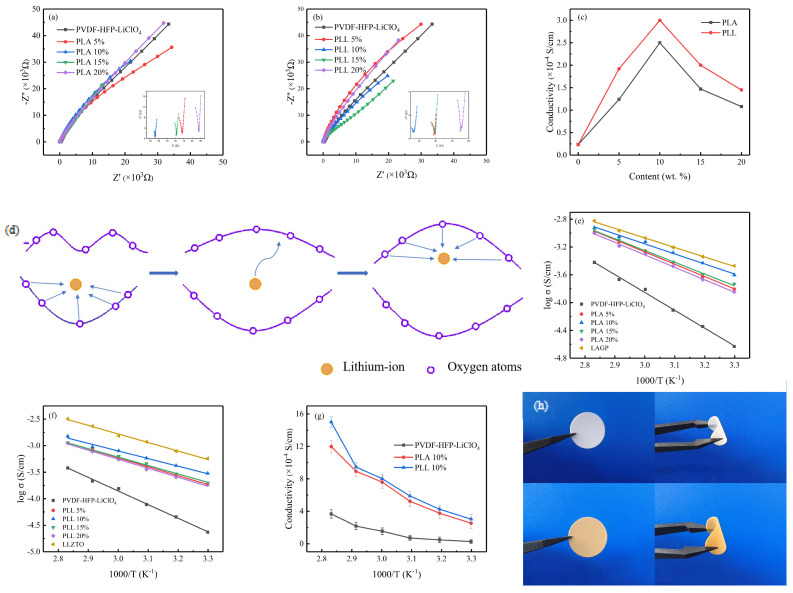
(**a**,**b**) Nyquist plot of SS/GPEs/SS battery tests at room temperature (inset: enlarged impedance spectra). (**c**) Ionic conductivity of GPEs with different LAGP or LLZTO content. (**d**) Schematic illustration for the proposed ion transport in gel polymers electrolytes. (**e**,**f**) Arrhenius plot for the GPEs as a function of the different temperatures. (**g**) Ionic conductivity values of PVDF−HFP−LiClO_4_, PLA 10%, and PLL 10% at different temperatures. (**h**) Flexibility demonstration of two bendable PLA 10% and PLL 10% membranes.

To find the right amount of addition at 0, 5, 10, 15, and 20 wt. %, blended membranes with different addition amounts were prepared according to the scheme in Section 2.2, and the level of ionic conductivity preliminarily judged the optimal addition amount of mixed membranes. The solution resistances (Rs) of different amounts of LAGP or LLZTO are shown in Figure 2a,b. As shown in Figure 2a, the bulk resistances of GPEs are 68 Ω, 36 Ω, 61 Ω, and 87 Ω when the addition amounts are 5%, 10%, 15%, and 20% of LAGP, while the bulk resistances of GPEs are 39 Ω, 27 Ω, 40 Ω, and 55 Ω when the addition amounts are 5%, 10%, 15%, and 20% of LLZTO, which is much smaller than that of PVDF-HFP-LiClO_4_ (up to 300 Ω). The schematic diagram of the mechanism is shown in Figure 2d. The addition of LAGP and LLZTO forms many oxygen vacancies, and the oxygen vacancies can be used as Lewis acid sites to accelerate the dissociation of lithium-ions, thereby improving the ionic conductivity. The ionic conductivity of each addition of LAGP or LLZTO is shown in Figure 2c, and the highest ionic conductivity of 2.5 × 10^−4^ S cm^−1^ and 3 × 10^−4^ S cm^−1^ is shown when LAGP and LLZTO are both added at 10%. In particular, according to Figure 2e,f, the activation energies of PVDF-HFP-LiClO_4_, PLA 10%, and PLL 10% membranes are 0.53 eV, 0.34 eV, and 0.31 eV. Figure 2g shows the ionic conductivity values of PVDF-HFP-LiClO_4_, PLA 10%, and PLL 10% membranes at different temperatures. They confirm that the highest ionic conductivity is obtained by adding 10% content of LAGP and LLZTO. Analyzing the results, when LAGP or LLZTO concentration is low, the GPEs cannot form an uninterrupted osmotic network and the ions are first transported in the polymer substrate. With the increase in the amount of LAGP/LLZTO, the lithium-ion transport pathway changes from the polymer substrate to the osmotic network phase so that it has a higher ionic conductivity. When the addition amount is more than 10%, the possible accumulation of too much LAGP/LLZTO destroys the osmotic network phase, resulting in a gradual decrease in ionic conductivity. Ta-doped LLZTO, on the other hand, has a cubic phase, which can provide more ion channels and can give it a higher ionic conductivity. As shown in Figure 2h, the upper part is the GPEs with 10% LAGP, and the lower half is the GPEs with 10% LLZTO. GPEs show good flexibility, such as bendability, presence, and processing properties. 

**Figure 3:**
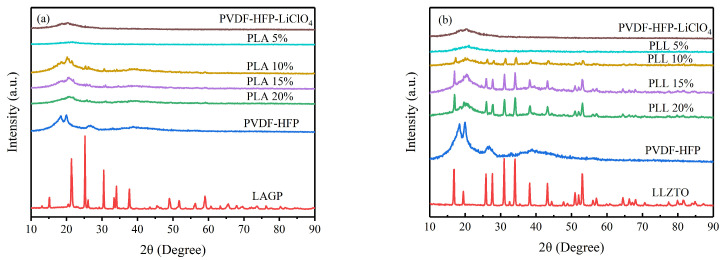
(**a**,**b**) XRD patterns of GPEs with various components.

As a complementing result, the XRD patterns of the different additions of LAGP and LLZTO are shown in Figure 3a,b, with the PVDF-HFP-LiClO_4_ matrix having two distinct characteristic peaks around 18° and 20° at 2θ. In contrast, adding LAGP/LLZTO broadens and suppresses these peaks. It is shown that the crystallinity decreases, and the amorphous phase content increases. The reduced crystallinity may be due to the random mixing of the matrix with LAGP/LLZTO, which introduces topological disorder and increases its ionic conductivity. When LAGP or LLZTO is added at 10%, the diffraction peaks of the matrix and LAGP/LLZTO in GPEs are almost identical, and the mixing is better than that of other films. Therefore, we chose this proportion of GPEs for subsequent studies.

**Figure 4:**
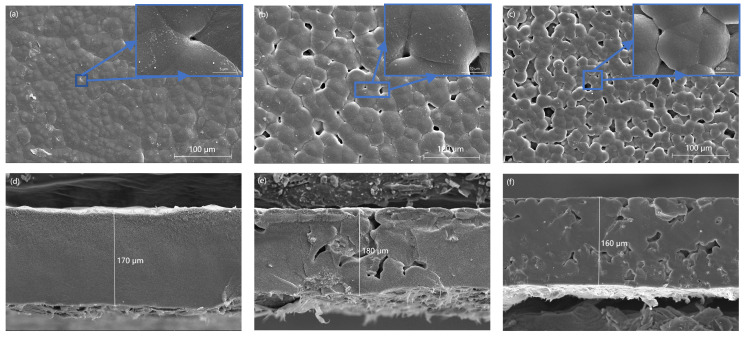
(**a**,**d**) The SEM surface and cross-section of PVDF-HFP-LiClO_4_ membrane. (**b**,**e**) The SEM surface and cross-section of PLA 10% membrane. (**c**,**f**) The SEM surface and cross-section of PLL 10% membrane.

Figure 4 shows SEM images of the surfaces and cross-sections of the PVDF-HFP-LiClO_4_ membrane, PLA 10% membrane, and PLL 10% membrane. The SEM plot of the surface of the PVDF-HFP-LiClO_4_ film is shown in Figure 4a, showing a dense and smooth surface, which is similar to the results published in the literature, while the cross-sectional plot (shown in Figure 4d) shows a thickness of about 170 μm. The PLA 10% film surface and cross-section SEM plots are shown in Figure 4b,e, and the PLL 10% film surface and cross-section SEM plots are shown in Figure 4c,f, with a thickness of approximately 180 μm for the PLA 10% film and a thickness of roughly 160 μm for the PLL 10% film. According to the surface diagram, the surface of the two films is relatively smooth, with some wrinkles and protrusions caused by the change in stress inside the film. As the polymer substrate and the LAGP/LLZTO penetrate each other (and previously had absorbed DMF when the film was dried in the drying oven), most of the DMF is evaporated, leaving behind pits that allow the film to absorb more of the liquid electrolyte and thus better transport lithium ions. In particular, according to the surface diagram, the PLL 10% film has more pits per unit area than the PLA 10% film, and, according to the cross-sectional diagram, the pit distribution of the PLL 10% film is more petite and more uniform than that of the PLA 10% film, which is more conducive to the transport of lithium ions. 

**Figure 5:**
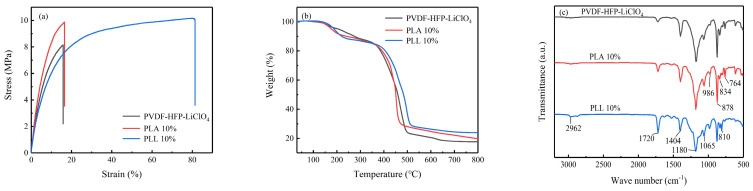
(**a**) Stress–strain curves of PVDF-HFP-LiClO_4_, PLA 10%, and PLL 10% membranes. (**b**) TGA curves of the PVDF-HFP-LiClO_4_, PLA 10%, and PLL 10% membranes. (**c**) FTIR spectra of the PVDF-HFP-LiClO_4_, PLA 10%, and PLL 10% membranes.

The mechanical stability of a battery is mainly dependent on the electrolyte membrane. When the internal stress of the battery exceeds the strength of the electrolyte membrane, different mechanical failures will occur, which will seriously affect the electrochemical performance of the battery [34]. As shown in Figure 5a, the tensile strength of the PVDF-HFP-LiClO_4_ membrane is 8.15 MPa, while that of the PLA 10% membrane is 9.88 MPa and that of the PLL 10% membrane is 10.16 MPa (higher than those of PVDF-HFP- LiClO_4_), indicating that the addition of LAGP/LLZTO can help increase the tensile strength of the electrolyte membrane. Although the tensile strength of PLA 10% film and PLL 10% film is similar, PLL 10% film has more significant strain than PLA 10% film (81.4% vs. 16.5%), indicating that PLL 10% film has more muscular toughness to resist lithium dendrite puncture. The perfect mechanical strength of the electrolyte membrane can keep the battery running for a long time without mechanical failure, making it safer.

The thermal stability of the electrolyte is an essential factor affecting the safety of the battery [35]. Figure 5b shows the comparative thermogravimetric analysis (TGA) curves for PVDF-HFP-LiClO_4_ membranes, PLA 10% membranes, and PLL 10% membranes. PVDF-HFP-LiClO_4_ membranes and PLA 10% membranes had no significant weight loss before 100 °C, while PLL 10% membranes had no consequential weight loss before 130 °C. Only 1% of the liquid content was lost for the three membranes. With the increase in temperature, the weight loss occurred at about 100–370 °C for PVDF-HFP-LiClO_4_ film and PLA 10% film (the PVDF-HFP-LiClO_4_ film was due to the decomposition of DMF, ETPTA, and PUA; the PLA 10% film was due to the decomposition of DMF, ETPTA, and PUA), and the weight loss of PLL 10% film occurred at about 130–390 °C, which was due to the decomposition of DMF, ETPTA, and PUA. The following weight loss of the PVDF-HFP- LiClO_4_ membrane occurred at nearly 370–500 °C, the subsequent weight loss of the PLA 10% membrane occurred at about 370–470 °C, and the next weight loss of the PLL 10% membrane appeared at about 390–510 °C. At 800 °C, PLL 10% membranes had more residual weight than PVDF-HFP-LiClO_4_ membranes and PLA 10% membranes (23.89% vs. 17.65% 19.74%). The results show that the addition of LAGP/LLZTO can help stabilize the polymer backbone, and all the cubic phases of Ta-doped LLZTO are more conducive to stabilizing the polymer backbone; in a sense, the thermodynamic properties of PLL 10% film can meet the application requirements of most batteries.

To investigate whether the polymer matrix and LAGP/LLZTO were fully polymerized, Figure 5c shows the FTIR spectra of a PVDF-HFP-LiClO_4_ membrane, a PLA 10% membrane, and a PLL 10% membrane. All three membranes were 2962 (C−H stretching), 1720 (C=O stretching), 1404 (C−H bending in CH_2_), 1180 (CF3 symmetrical stretching), 1065 (C−C stretching), 986 and 764 (crystalline phase), 878 (C−C skeletal vibration), 834 (C−H bending in CH_2_), and 810 cm^−1^ (CF3 stretching). This indicated that all components were successfully incorporated into the membrane matrix.

**Figure 6:**
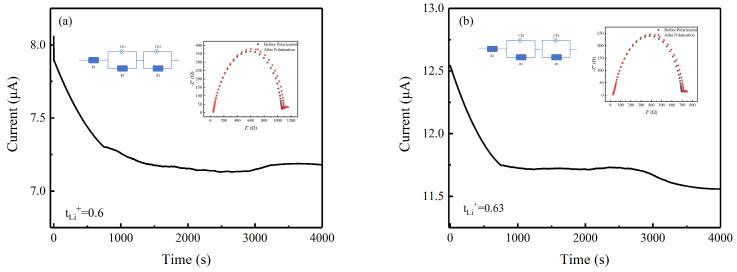
(**a**,**b**) DC polarization curve of the Li/PLA 10%/Li and Li/PLL 10%/Li batteries at a polarization voltage of 10 mV (inset: the simulating equivalent circuit and impedance spectra of the symmetric battery before and after polarization).

The lithium-ion transfer number (*t*_*Li*_^+^) is another key factor affecting the suitability of any electrolyte membrane. Table 1 lists the *t*_*Li*_^+^ values for PLA 10% films and PLL 10% films, and Figure 6a,b show their corresponding AC impedance spectra and DC polarization curves, with R1 representing the electrolyte resistance, R2 and R3 being the electrolyte–electrode interface resistances, and CE1 and CE2 being the two constant phase elements. The simulated impedance response was directly calculated using ZView 3.1 software. The *t*_*Li*_^+^ values of PLA 10% membranes and PLL 10% membranes were 0.6 and 0.63, respectively. The increase in the number of lithium ions after the addition of the LAGP/LLZTO may have been due to the fact that the functional groups in the LAGP/LLZTO complex with the polymer to form a semi-interpenetrating structure, which allowed the lithium ions to be transported rapidly, thereby eliminating the concentration polarization.

**Figure 7:**
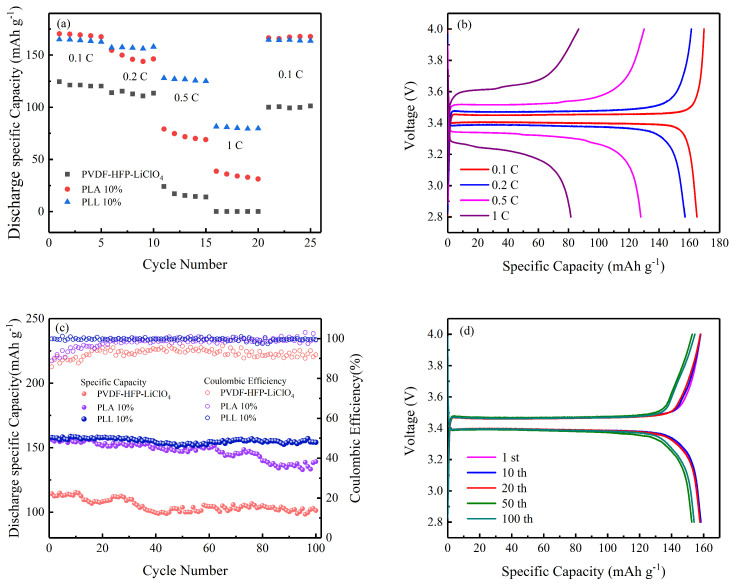
(**a**) Rate capability of PVDF−HFP−LiClO_4_−based LFP/Li, PLA 10%−based LFP/Li, and PLL 10%−based LFP/Li batteries. (**b**) Galvanostatic charge−discharge curves of PLL 10%−based LFP/Li battery at 0.1, 0.2, 0.5, and 1 C. (**c**) Cycling performances of PVDF−HFP−LiClO_4_−based LFP/Li, PLA 10%−based LFP/Li, and PLL 10%−based LFP/Li batteries. (**d**) Galvanostatic charge−discharge curves of PLL 10%−based LFP/Li battery at 0.2 C for 1 cycle, 10 cycles, 20 cycles, 50 cycles, 100 cycles.

To reveal the electrochemical properties of PVDF-HFP-LiClO_4_ membranes, PLA 10% membranes, and PLL 10% membranes, LFP/GPEs/Li batteries were assembled in a CR2025 battery case in an argon-filled glove box and tested in an incubator at 25 °C. Figure 7a illustrates the specific discharge capacity of PVDF-HFP-LiClO_4_, PLA 10%, and PLL 10% batteries at different current densities. At 0.1 C, 0.2 C, and 0.5 C, the discharge specific capacity of PVDF-HFP-LiClO_4_ battery was 124, 114, and 24 mAh g^−1^ in the first circle, respectively. When it reached 1 C, the discharge specific capacity was reduced to 0 mAh g^−1^. When the rate was restored to 0.1 C, the discharge specific capacity could only be restored to 80.6% of the original discharge specific capacity, which was 100 mAh g^−1^. There was little difference in discharge-specific capacity between PLA 10% and PLL 10% batteries at 0.1 C and 0.2 C current density, but there was a significant difference between 0.5C and 1C current densities (0.5 C: 79 vs. 128 mAh g^−1^; 1 C: 81.5 vs. 38 mAh g^−1^). After restoring to 0.1 C, the discharge specific capacity of the two batteries quickly recovered. Figure 7b shows the charge–discharge curves of a PLL 10% battery at each current density. At a rate of 0.1 C, the battery had a charging platform and a discharge platform under the reversible redox potential of about 3.45 V and 3.4 V, and the polarization voltage also increased with the increase in the current density. Experiments showed that PLL 10% film had a better rate of performance than the other two films.

To further test the long-term cycling stability of the battery, the three membrane-assembled batteries were cycled at 25 °C at a rate of 0.2 C, with a cut-off voltage range of 2.8 V–4 V. Figure 7c shows the long-term cycling stability of the three batteries, and Figure 7d shows the charge–discharge curves for the 1st, 10th, 20th, 50th, and 100th turns of the battery assembled with the PLL 10% film. After 100 cycles, the discharge-specific capacity of PVDF-HFP-LiClO_4_ batteries decreased from 114 mAh g^−1^ to 101 mAh g^−1^, the capacity retention rate was 88.6%, and the average coulombic efficiency was 92.7%. There was no significant difference in the initial discharge specific capacity of PLA 10% and PLL 10% batteries, but, after 100 cycles, PLL 10% batteries had a higher discharge specific capacity (154 mAh g^−1^ vs. 139 mAh g^−1^) and a higher average coulombic efficiency (99.7% vs. 98.2%). PLL 10% batteries had a slight upward trend in discharge-specific capacity after 60 cycles, which could be the formation of a stable SEI film. These results show that, in PLL 10% batteries, the addition of LLZTO to the polymer is beneficial to the increase in amorphous regions and the formation of functionalized cross-linked structures, which is conducive to the cycling stability of the batteries.

## 4. Conclusions

In summary, in this paper, GPEs were designed and synthesized by a UV curing process, and the effects of no filler or filler (LAGP and LLZTO) on GPEs were compared. The experimental results show that GPEs with LLZTO have higher ionic conductivity than PVDF-HFP-LiClO_4_ and PLA 10% batteries (3 × 10^−4^ vs. 2.34 × 10^−4^ vs. 2.5 × 10^−4^ S cm^−1^) and a higher lithium-ion transfer number (0.63 vs. 0.6) than PLA 10% batteries. Assembled into LiFePO_4_/GPEs/Li batteries, PLL 10% batteries have a higher discharge specific capacity than PVDF-HFP-LiClO_4_ and PLA 10% batteries at 1C current density (81.5 vs. 0 vs. 38 mAh g^−1^). After 100 cycles of cycling at 0.2C, PLL 10% batteries have a higher capacity retention than PVDF-HFP-LiClO_4_ and PLA 10% batteries (98.1% vs. 88.6% vs. 89.1%) at 25 °C. Therefore, the obtained GPEs have good thermal stability and 100-cycle stability, indicating that the electrolyte membrane prepared by this economical, efficient, and simple synthesis strategy is a very promising candidate material for lithium batteries. 

## Figures and Tables

**Table 1:** Experimentally measured parameters of solid electrolytes and their lithium-ion transfer numbers (*t*_*Li*_^+^) calculated at room temperature.

Gel Polymer Electrolytes	I_0_ (μA)	I_s_ (μA)	R_0_ (Ω)	R_s_ (Ω)	t_Li_^+^
PLA 10%	8.11	7.17	1025.462	1058.558	0.6
PLL 10%	12.79	11.568	671.729	691.406	0.63

## Data Availability

Date are contained within the article.
